# Kynurenine Promotes Phosphate-Induced Endothelial Calcification via Endothelial-to-Mesenchymal Transition, Osteoblastic Differentiation and AhR Activation

**DOI:** 10.3390/toxins17080421

**Published:** 2025-08-19

**Authors:** Martina Molinaro, Mario Cozzolino, Paola Ciceri

**Affiliations:** Laboratory of Experimental Nephrology, Department of Health Sciences, University of Milan, 20142 Milan, Italy

**Keywords:** kynurenine, phosphate, calcification, endothelial cells, AhR, EndMT, osteoblastic differentiation

## Abstract

In end-stage renal disease (ESRD), the accumulation of solutes normally excreted by the kidneys contributes to multiple complications, including vascular calcification (VC), a key factor in the heightened cardiovascular risk seen in these patients. Among VC drivers, hyperphosphatemia and the uremic milieu are major contributors. Kynurenine, a tryptophan metabolite classified as a uremic toxin, may further exacerbate this process. This study investigated whether kynurenine amplifies high phosphate (Pi)-induced calcification in human aortic endothelial cells (HAEC). Cells were treated with Pi and kynurenine for up to seven days. Kynurenine increased Pi-induced calcium deposition by 36%, accompanied by enhanced endothelial-to-mesenchymal transition (EndMT) and osteoblastic differentiation. Mechanistically, kynurenine activated the aryl hydrocarbon receptor (AhR) pathway, and pharmacological inhibition of AhR partially attenuated this effect. These findings suggest that kynurenine contributes to VC in ESRD by potentiating phosphate-induced endothelial dysfunction via AhR signaling.

## 1. Introduction

Kidney failure leads to the accumulation of various solutes that are normally excreted or metabolized by the kidneys, contributing to the symptoms characteristic of end-stage renal disease (ESRD). These retained compounds, collectively known as uremic toxins, form a heterogeneous group that contributes to the uremic milieu—an environment that accelerates pathological processes. Uremic toxins vary in size and molecular weight and are typically classified into three groups: small water-soluble molecules, middle molecules, and protein-bound toxins. Among the protein-bound toxins, tryptophan-derived metabolites, including kynurenine (Kyn), are significantly elevated in ESRD. These compounds can activate the aryl hydrocarbon receptor (AhR) in the vasculature, promoting oxidative stress and driving pathological processes such as inflammation and cardiovascular disease [1,2].

Cardiovascular complications are the leading cause of morbidity and mortality in ESRD patients, with vascular calcification (VC) playing a central role. VC increases arterial stiffness, leading to elevated pulse wave velocity and blood pressure, which in turn contribute to left ventricular hypertrophy and heart failure [3,4,5]. While VC in ESRD primarily affects the tunica media, recent studies have shown that high phosphate (Pi) levels and uremic serum can also induce calcification of the endothelial extracellular matrix, suggesting a broader impact on vascular integrity [6,7,8,9].

In a previous study, we demonstrated that a specific dialysis membrane could reduce the pro-calcifying effect of uremic serum. This benefit was associated with improved clearance of certain uremic toxins, including tryptophan metabolites such as Kyn, likely due to a modest reduction in protein-bound toxins [10]. Building on these findings, the aim of the present study was to investigate whether kynurenine exerts a pro-calcifying effect in an in vitro model of endothelial calcification.

## 2. Results

### 2.1. Effect of Kynurenine on Pi-Induced Endothelial Calcification

**Figure 1:**
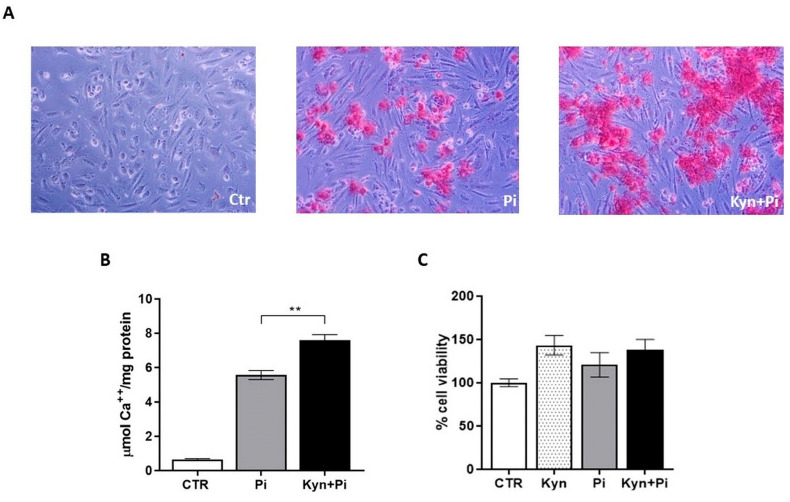
Kynurenine effect on calcification. (**A**) Representative images of Alizarin Red staining of HAEC treated with 3 mM Pi and 30 μM Kyn after 7 days. Magnification 100×. (**B**) Calcium deposition is measured by de-staining and normalized by protein content expressed as μmol Ca^++^/mg protein. (**C**) Effect of Kyn on cell viability, evaluated by MTT-Assay. Data are presented as the mean ± SEM of at least three experiments in triplicate. ** *p* < 0.01.

Pre-treatment with Kyn for 5 days followed by co-stimulation by Kyn+Pi exacerbated Pi-induced calcification, as shown by an increase in the number and size of calcium crystal formation (Figure 1A). Calcium measurement demonstrated a significant increase in calcium deposition by Kyn+Pi compared to Pi alone of 36% (Pi 5.56 ± 0.26 vs. Kyn+Pi 7.59 ± 0.32; μmol Ca^++^/mg protein; ** *p* < 0.01; Figure 1B). The treatment with Kyn, even when combined with Pi, did not significantly affect cell viability, as shown by MTT-Assay (Figure 1C).

### 2.2. Effect of Kynurenine on Pi-Induced Endothelial-to-Mesenchymal Transition

**Figure 2:**
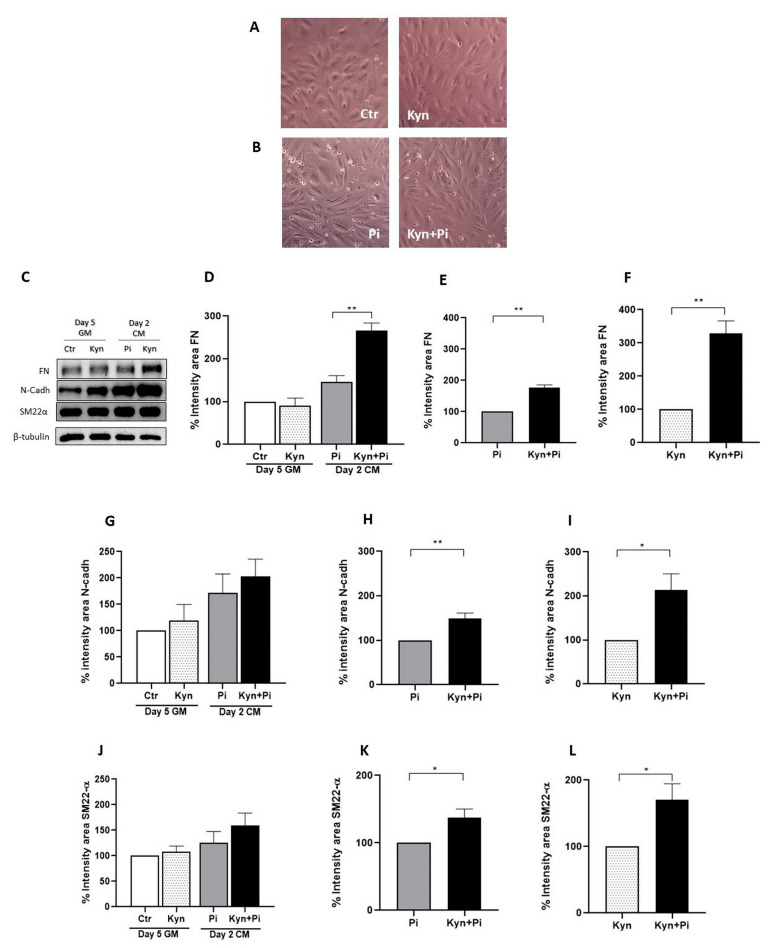
Kynurenine effect on endothelial-to-mesenchymal transition. (**A**) Representative images of HAEC treated with 30 μM Kyn for 5 days in growth medium (GM) or (**B**) for an additional 2 days with 3.5 mM Pi or Kyn+Pi in calcification medium (CM). Magnification 100×. (**C**–**L**) Protein expression of mesenchymal markers after 5 days in GM and after treatment with 3.5 mM Pi for an additional 2 days in CM. (**C**) Representative bands of FN, N-Cadh and SM22α and the housekeeping β-tubulin. (**D**–**F**) FN relative intensity area, with respect to control (**D**), Pi (**E**) and Kyn (**F**). (**G**–**I**) N-Cadh relative intensity area, with respect to control (**G**), Pi (**H**) and Kyn (**I**). (**J**–**L**) SM22α relative intensity area, with respect to control (**J**), Pi (**K**) and Kyn (**L**). Data are presented as the mean ± SEM of at least three experiments in triplicate. * *p* < 0.05, ** *p* < 0.01.

Treatment with Kyn induced a phenotypical change of endothelial cells from cobblestone to spindle-shaped, already evident after 5 days of treatment in growth medium (GM) (Figure 2A). Also, treatment with Pi induced a spindle shape phenotypical change on day 2 of calcification medium (CM) that was maintained in the co-treated Kyn+Pi endothelial cells (Figure 2B). The change in cellular shape is a sign of endothelial-to-mesenchymal transition (EndMT); therefore, we investigated the expression of mesenchymal markers after treatment with Kyn and Pi. We found that following the 5 days of Kyn treatment in GM, there was no modification in fibronectin (FN) levels compared to control. However, after two days of Pi treatment, Pi induced an increased expression of FN, which was further increased by the combination of Kyn+Pi (Figure 2C,D). Evaluating the effect of the combination of Kyn+Pi with respect to both Pi and Kyn alone, we found a significant effect (176.6 ± 9.2 and 328.7 ± 37.8; % increase; Kyn+Pi vs. Pi and Kyn, respectively, ** *p* < 0.01, Figure 2E,F). The same trend has been found for both N-cadherin (N-Cadh) and Smooth muscle 22 α (SM22-α). N-Cadh was not affected by 5 days of Kyn treatment in GM but was induced by Pi alone and the combination of Kyn+Pi (Figure 2G). Also for N-Cadh, the combination of Kyn+Pi induced a significant increase with respect to both Pi and Kyn alone (149.2 ± 8.9 and 213.3 ± 32.5; % increase; Kyn+Pi vs. Pi and Kyn respectively, ** *p* < 0.01, * *p* < 0.05, Figure 2H,I). Also, SM22-α was not affected by Kyn treatment in GM but increased after Pi and the combination of Kyn+Pi treatment (Figure 2J). Even if less pronounced, respect FN and N-Cadh, also for SM22α Kyn+Pi induced a significant increase with respect to both Pi and Kyn alone (137.8 ± 12.4 and 170.9 ± 20.7; % increase; Kyn+Pi vs. Pi and Kyn respectively, * *p* < 0.05, Figure 2K,L).

### 2.3. Effect of Kynurenine on Pi-Induced Migratory Capability

**Figure 3:**
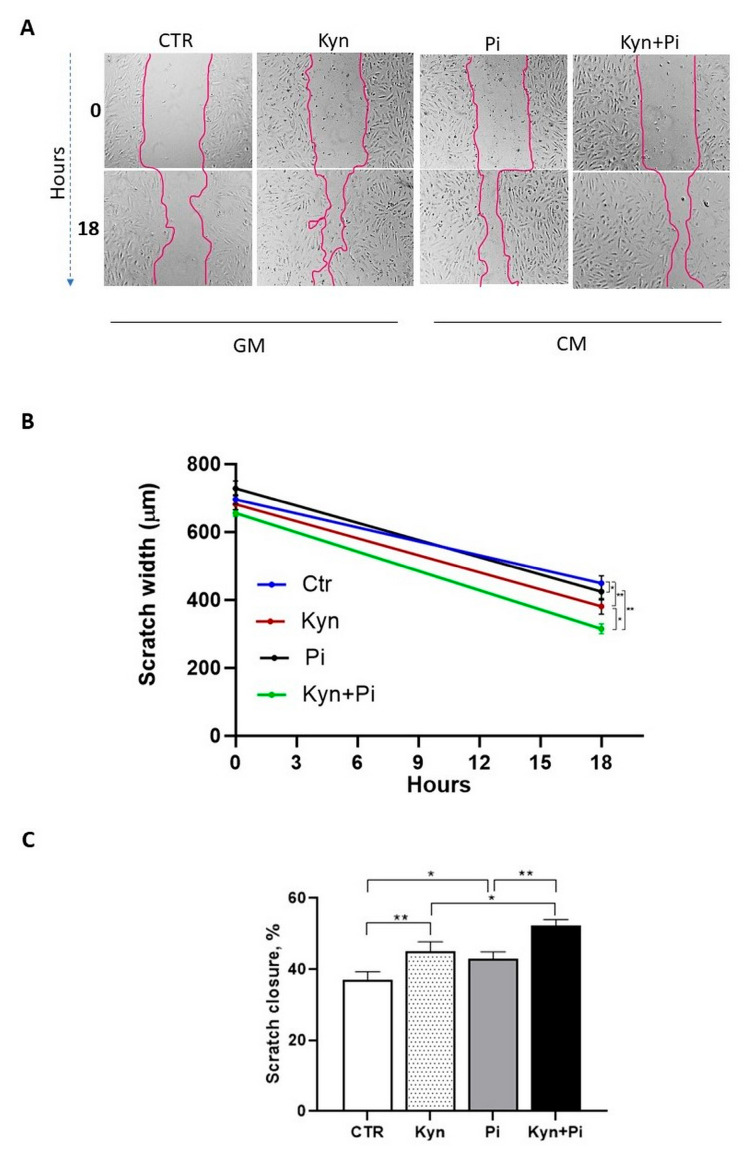
Kynurenine effect on migratory properties. Migration was evaluated from T_0_ to 18 h. (**A**) Representative images of scratches in control and 30 μM Kyn after 5 days of treatment in GM and an additional 2 days of treatment with 3 mM Pi in CM. Magnification 40×. (**B**) Scratches width in the four different groups. (**C**) Quantification of scratch closure expressed as a percentage. Data are presented as the mean ± SEM of five experiments in triplicate. * *p* < 0.05, ** *p* < 0.01.

A functional consequence of EndMT is the acquisition of migratory properties. We therefore performed the scratch wound healing assay (Figure 3A,B) that revealed that between day 2 and 3 after calcification medium switch, 18 h after the scratch was made, HAEC treated with Kyn exhibited significantly enhanced migratory capacity compared to the controls (Kyn 45.2 ± 2.8; Ctr 36.9 ± 2.6; scratch closure %; ** *p* < 0.01; Figure 3C). Also, Pi treatment induced an increase in migratory properties (Pi 42.9 ± 2.2; scratch closure %; * *p* < 0.05 Figure 3C). Interestingly, combined treatment of Kyn with Pi further increased HAEC migratory capabilities with a significant increase in closure, both with respect to Kyn and Pi alone (Kyn+Pi 52.3 ± 1.9; scratch closure %; * *p* < 0.05, ** *p* < 0.01; Figure 3C).

### 2.4. Effect of Kynurenine on Pi-Induced Osteoblastic Differentiation

**Figure 4:**
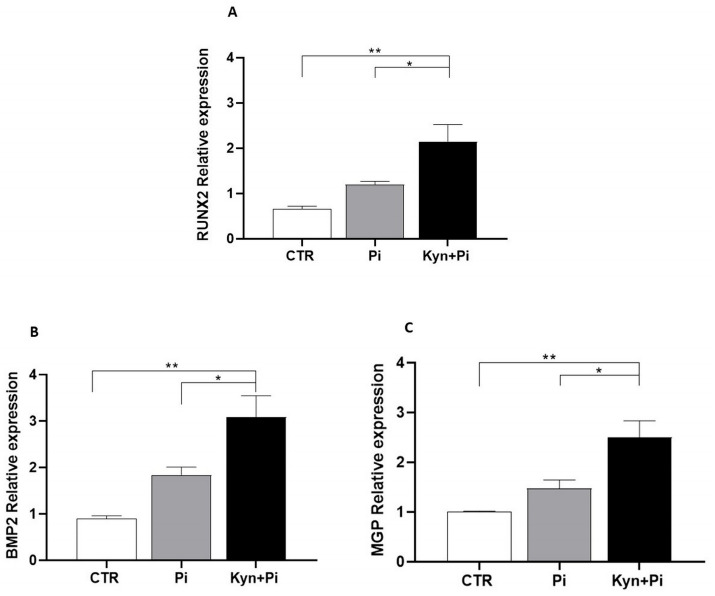
Kynurenine effect on endothelial osteoblastic differentiation. Effect of 3.5 mM Pi and 30 μM Kyn for 7 days on mRNA relative expression of the master gene of the osteoblastic cascade RUNX2 (**A**) and of the osteoblastic markers BMP2 (**B**) and MGP (**C**). * *p* < 0.05, ** *p* < 0.01.

To evaluate whether the kynurenine-induced increase in Pi-dependent calcium deposition was due to increased osteoblastic differentiation, we measured mRNA levels of the master gene of the osteoblastic cascade, RUNX2. We found that the co-treatment of Kyn+Pi was able to significantly increase RUNX2 expression compared to Pi after 7 days of co-stimulation (2.14 ± 0.37 vs. 1.19 ± 0.07; Kyn+Pi vs. Pi; RUNX2; rel.exp; * *p* < 0.05; Figure 4A). Also, the osteoblastic markers BMP2 and MGP showed a significant increase in co-treated Kyn+Pi HAEC compared with Pi alone (3.08 ± 0.45 vs. 1.84 ± 0.16; 2.5 ± 0.33 vs. 1.48 ± 0.16; Kyn+Pi vs. Pi; BMP2, MGP, respectively; rel.exp; * *p* < 0.05; Figure 4B,C), supporting the evidence of an increased osteoblastic differentiation induced by the combination of Kyn and Pi.

### 2.5. Effect of Co-Stimulation with Kynurenine and Pi on AhR Pathway

**Figure 5:**
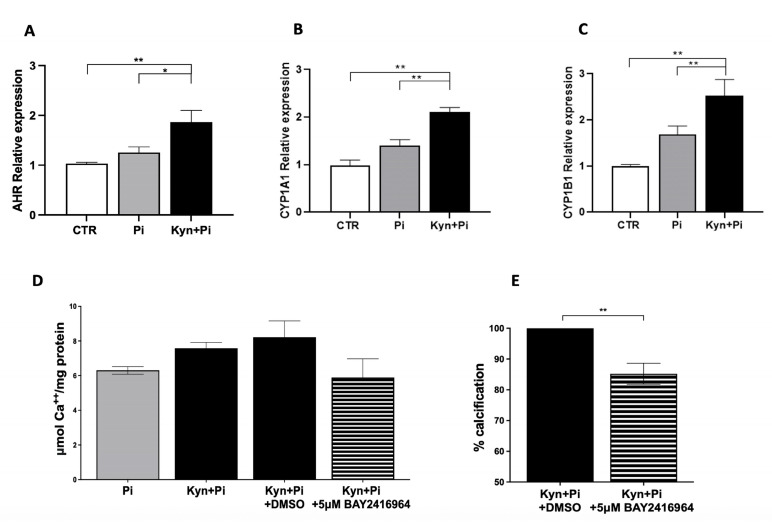
Kynurenine effect on the AhR pathway. Effect of 3.5 mM Pi and 30 μM Kyn for 7 days on mRNA relative expression of the transcription factor AhR (**A**) and the downstream genes CYP1A1 (**B**) and CYP1B1 (**C**). (**D**,**E**) Effect of the AhR antagonist BAY2416964 (5 μM) on calcium deposition induced by 3 mM Pi and 30 μM Kyn after 7 days. (**D**) μmol Ca++/mg protein of one representative experiment; (**E**) percentage of calcification in cells treated with BAY2416964 compared with cells treated with the vehicle DMSO at the same concentration; mean of 5 experiments. * *p* < 0.05, ** *p* < 0.01.

Since Kyn is known to signal through the aryl hydrocarbon receptor (AhR) and the downstream molecules CYP1A1 and CYP1B1, we next investigated their expression by RT-PCR. As shown in Figure 5, the co-stimulation of Kyn+Pi induced a significant increase in AhR paralleled by an increase in both CYP1A1 and CYP1B1 after 7 days (1.86 ± 0.2 vs. 1.25 ± 0.11; 2.11 ± 0.09 vs. 1.40 ± 0.12; 2.52 ± 0.35 vs. 1.68 ± 0.18; Kyn+Pi vs. Pi; AhR, CYP1A1, CYP1B1 respectively; rel.exp; ** *p* < 0.01, * *p* < 0.05, Figure 5A–C). Interestingly, the addition of the AhR antagonist BAY 2416964 at a concentration of 5 µM was able to partially prevent Kyn-induced increase in calcium deposition (Figure 5D) with a significant decrease in calcification of 14.8% (Figure 5E), demonstrating the relevance of AhR pathway activation to the calcification process.

## 3. Discussion

Vascular calcification (VC) is caused, in end-stage renal disease (ESRD), by the retention of deregulated levels of uremic toxins due to the impairment of kidney function. VC causes arterial stiffness that is relevant for the development of cardiovascular comorbidities in ESRD and is associated with increased morbidity and mortality in these patients [11,12,13]. Among several uremic factors, high-phosphate (Pi) levels play a central role in the pathogenesis of VC, together with a comparably important role played by the other components of the uremic milieu [14].

Vascular calcification (VC) is typically studied in vitro using vascular smooth muscle cells (VSMC) exposed to high phosphate (Pi), as the tunica media is the key vascular layer responsible for arterial stiffness. However, endothelial cells—forming the interface between blood and the vessel wall—play a critical regulatory role in vascular tone. Endothelial dysfunction, often triggered by the uremic milieu, is increasingly recognized as an active contributor to vascular dysregulation in ESRD and is considered a ‘non-traditional’ risk factor for cardiovascular comorbidities [15]. Recently, our group demonstrated the ability of endothelial cells to calcify when challenged with high-Pi [6]. In the same study, it has also been demonstrated that uremic serum is able to induce endothelial extracellular matrix calcification. Interestingly, there is a linear correlation between the uremic serum pro-calcifying effect on endothelial cells and VSMC, supporting a role for the uremic milieu in the calcification of the entire vessel. Endothelial calcification can be assumed as an extreme form of endothelial dysfunction, and the pathological significance may lie in the fact that endothelial calcification may provide osteogenic precursors that migrate into the tunica media and participate in media calcification itself. In fact, data have been published in two different transgenic animal models of VC, such as a mouse model with gene deletion for matrix Gla Protein and Ins2Akita/+ mice, a model of diabetes [16]. In these models, the authors demonstrated the presence of osteogenic cells of endothelial origin in the media layer of calcified vessels. Therefore, identifying which uremic toxins contribute to endothelial dysfunction and the acquisition of osteogenic features may aid in developing targeted strategies to reduce the pro-calcific potential of uremic serum. In this study, we focused on kynurenine (Kyn), a biologically active metabolite of tryptophan and a recognized uremic toxin. Kynurenine levels are increased in chronic kidney disease not only as a consequence of renal impairment but also due to modifications in tryptophan metabolism [17].

Experimental evidence demonstrated that kynurenine circulating levels associate with incident cardiovascular disease and all-cause mortality in the elderly and in patients with stable angina [18,19]. Moreover, recently it has been demonstrated that Kyn serum levels associate with cardiovascular disease comorbidities and overall mortality in a large group of patients with chronic kidney disease [20]. A relationship between Kyn and endothelial dysfunction has been suggested by some studies that found a link between increased circulating Kyn levels and markers of endothelial dysfunction [21,22,23]. In line with the supposed involvement of Kyn in the development of cardiovascular disease and in the endothelial dysfunction process, together with our previous study on uremic serum pro-calcifying effect [10], in this study, we demonstrated a Kyn exacerbating effect on Pi-induced endothelial calcification and transformation. In the model of endothelial cells treated with high-Pi, in the first days of treatment, endothelial-to-mesenchymal transition (EndMT) occurs, followed by osteoblastic differentiation [6]. To study the effect of Kyn, we utilized a concentration of 30 µM that is in the same range as the highest concentration of total Kyn measured in uremic patients [20], thus mimicking in vitro the effect on endothelium likely exerted in vivo. At this concentration, Kyn is able to exacerbate Pi-induced EndMT as demonstrated by an increased expression of mesenchymal markers such as fibronectin, N-Cadherin and SM22α. Supporting the transition to a mesenchymal phenotype, there is also a change to a spindle shape and the acquisition of migratory capability. Thus, kynurenine may promote the migration of mesenchymal-like cells of endothelial origin into the tunica media, where the pro-osteogenic environment could further drive their differentiation into osteoblast-like cells. Additionally, our study demonstrated that kynurenine in combination with Pi exacerbates endothelial calcification, as evidenced by increased calcium deposition and enhanced osteoblastic-like differentiation, which was not affected by kynurenine alone. Our group recently demonstrated that endothelial calcification may be relevant in ESRD patients since in arteries from uremic patients there is deposition of intracytoplasmic fine calcium phosphate crystals that, together with osteoblastic differentiation, demonstrate the dramatic effect of the uremic milieu on endothelial features [6]. Our current findings, using an in vitro model of endothelial calcification, suggest that elevated kynurenine levels may contribute to uremia-induced endothelial dysfunction and calcification in ESRD patients. To explore the underlying mechanism by which kynurenine exacerbates Pi-induced EndMT and calcification, we investigated the aryl hydrocarbon receptor (AhR) pathway—a known target of kynurenine [24]. We found that in our model, Kyn and Pi have a combined effect significantly inducing an elevation in the expression of the transcription factor AhR, and of the downstream effectors CYP1A1 and CYP1B1. Supporting the hypothesis that the AhR pathway may be relevant to the endothelial calcification process, we found that the AhR antagonist BAY2416964 was able to partially prevent the worsening effect of Kyn on Pi-induced calcification. Therefore, in our endothelial cell model, the effects of kynurenine are mediated by AhR, which is also the known target of another tryptophan-derived uremic toxin, indoxyl sulfate, in promoting calcification and endothelial dysfunction in both valvular interstitial aortic cells and the endothelium [25,26,27]. Thus, our results support a role of Kyn in the onset of processes leading to cardiovascular comorbidities development, in line with others that, in different experimental settings, demonstrate an involvement of Kyn in abnormal angiogenesis and thrombosis [22,23].

In conclusion, this study demonstrates that kynurenine has an additive exacerbating effect on Pi-induced endothelial calcification. Our findings highlight the harmful contribution of the uremic milieu by isolating and elucidating the specific role of kynurenine in this process. These results underscore the importance of improving both dialytic strategies and pharmacological approaches to better manage uremic toxin burden, with the goal of reducing cardiovascular morbidity and mortality and improving the quality of life in patients with ESRD.

## 4. Materials and Methods

### 4.1. Endothelial Cell Culture and Induction of Calcification

Human Aortic Endothelial Cells (HAEC) were purchased from ATCC (ATCC, Manassas, VA, USA). They were sub-cultured on 0.1% *w*/*v* gelatin-coated plates and in growth medium (GM) (Vascular Cell Basal Medium (ATTC, #ATCC-PCS-100-030), 2% FBS, 100 U/mL penicillin, 0.1 mg/mL streptomycin supplemented with Endothelial Cell Growth Kit-BBE (#ATCC-PCS-100-040). HAEC were seeded, and when the culture was at full confluence, they were switched to calcification medium (CM) (DMEM high-glucose, 5% FBS, 10 mM sodium pyruvate, Endothelial Cell Growth Kit-BBE, as indicated by the supplier) and challenged with Na3P04 (Pi) 3 or 3.5 mM for 7 days.

Pre-treatment with 30 µM kynurenine (Kyn) started 5 days before the addition of 3 mM Pi for up to an additional 7 days in co-treatment with Pi; in experiments with the AhR antagonist, 5 µM BAY 2416964 (MedChemExpress, Monmouth Junction, NJ, USA) was added concomitantly with 30 µM kynurenine.

The medium was replaced every 2 days. Cells were used between the fifth and seventh passages.

### 4.2. Alizarin Red Staining and Quantification of Calcium Deposition by De-Staining

Quantification of calcium (Ca^++^) deposition was performed by Alizarin Red S staining, followed by perchloric acid (HClO_4_) de-staining. HAEC were fixed with 70% EtOH and stained with Alizarin Red 1 mg/mL solution for 1 h, and then they were de-stained for 24 h with 5% HClO_4_. Ca^++^ content was determined colorimetrically at 450 nm wavelength. Protein content was quantified by the BCA protein assay kit (#23225 ThermoFisher, Waltham, MA, USA), and Ca^++^ deposition was normalized on protein content and expressed as μmol Ca^++^/mg protein.

### 4.3. Cell Proliferation Assay

Cell viability was determined by MTT-Assay Kit Cell Proliferation (ab211091, Abcam, Cambridge, UK). Cells were seeded at a density of 50,000 cells/cm^2^ in 48-well plates, in duplicate of 100,000 cells/cm^2^ in 96-well plates, in triplicate. At day 7 of the calcification period with 3 mM Pi, the medium was discarded, and an equal amount of MTT-Reagent and serum-free medium was added. After 3 h of incubation at 37 °C, 300 μL or 150 μL of MTT-Solvent was added to each well and mixed on an orbital shaker for 15 min. The absorbance signal was measured at a wavelength of 590 nm.

### 4.4. Endothelial Cells Scratch Wound Healing Assay

At day 2 of Pi treatment, the scratch was performed with a sterile pipette tip, and pictures were acquired at T0 and after 18 h (T18). Cell migration was evaluated by quantifying the scratch width with the ToupView program and expressed as a percentage of scratch closure.

### 4.5. RNA Extraction and RT-PCR

Treated HAEC were collected with TRI-Reagent buffer, and total RNA was extracted using Direct-zol RNA MiniPrep (Zymo Research, #R2050, Irvine, CA, USA) according to the manufacturer’s instructions. After reverse transcription performed with a high-capacity RNA-to-cDNA kit (#4387406 ThermoFisher, Waltham, MA, USA), TaqMan PCR was conducted using a StepOne Real-Time PCR system. The reaction mixture (15 μL) contained 7.5 μL of 2X Taqman Universal PCR Master Mix, 0.75 μL of TaqMan gene assay (Hs01047973_m1 RUNX2, Hs00154192_m1 BMP2, Hs00179899_m1 MGP, Hs00169233_m1 AhR, Hs00153120_m1 CYP1A1, Hs00164383_m1 CYP1B1; ThermoFisher, Waltham, MA, USA), 30 ng for RUNX2 and BMP2, 11 ng for MGP and 50 ng for AhR, CYP1A1, CYP1B1 of the cDNA sample and water. Amplification of cDNA was normalized to simultaneous amplification of an internal housekeeping gene, β-actin, and calibrated to a low-expressing, normalized target sample.

### 4.6. Protein Extraction and Western Blotting

HAEC were harvested in ice-cold homogenization buffer (50 mM Tris pH 8 with 0.5% IGEPAL, 1 mM benzamidine HCl, 1 mM sodium fluoride, 10 mM β-glycerophosphate, 1 mM phenylmethanesulfonyl fluoride and complete protease inhibitor (#11836153001 Roche, Basil, Switzerland). Then the cells were subjected to 3 freeze-thawed cycles, sonication (5 × 20 s at 20% power) and centrifuged at 13,400× *g* for 15 min at 4 °C. Protein content was quantified as described above. Samples were denatured with Laemmli Buffer 5× and resolved on 7.5% SDS-PAGE (for N-Cadh and FN) and 12% (for SM22α) and then transferred to a PVDF membrane. After blocking with 5% dry milk in PBS-T or TBS-T (TBS pH 7.5 and 0.05% TWEEN-20), membranes were incubated overnight (O/N) at 4 °C with primary antibodies as follows: anti-N-cadherin (Abcam, ab18203; polyclonal rabbit, 1:1000), anti-fibronectin (FN; Abcam, ab2413; polyclonal rabbit, 1:500), and anti-SM22α (Abcam, ab10135; polyclonal goat, 1:6000). The following day, cells were incubated with the appropriate secondary antibodies. Protein bands were visualized using an ECL detection kit, and area intensity was measured by Image-Lab 6.0.1 software (Biorad, Hercules, CA, USA) and normalized on the β-tubulin band.

### 4.7. Statistical Analysis

Results are expressed as mean ± SEM of at least three experiments in triplicate. Differences between groups were analyzed by *t*-test, by one- and two-way ANOVAs and were considered statistically significant when *p* < 0.05.

## Data Availability

The original contributions presented in this study are included in the article. Further inquiries can be directed to the corresponding author.

## Abbreviations

The following abbreviations are used in this manuscript:

ESRD	End-stage renal disease
VC	Vascular calcification
EndMT	Endothelial-to-mesenchymal transition
Pi	High phosphate
Kyn	Kynurenine
FN	Fibronectin
N-Cadh	N-cadherin
SM22α	Smooth muscle 22 α
AhR	Aryl hydrocarbon receptor
